# Inhibition of Vanadium Cathodes Dissolution in Aqueous Zn‐Ion Batteries

**DOI:** 10.1002/adma.202310645

**Published:** 2024-01-23

**Authors:** Yuhang Dai, Chengyi Zhang, Jianwei Li, Xuan Gao, Ping Hu, Chumei Ye, Hongzhen He, Jiexin Zhu, Wei Zhang, Ruwei Chen, Wei Zong, Fei Guo, Ivan P. Parkin, Dan J. L. Brett, Paul R. Shearing, Liqiang Mai, Guanjie He

**Affiliations:** ^1^ Christopher Ingold Laboratory Department of Chemistry University College London London WC1H 0AJ UK; ^2^ State Key Laboratory of Advanced Technology for Materials Synthesis and Processing Wuhan University of Technology Wuhan 430070 China; ^3^ Electrochemical Innovation Lab Department of Chemical Engineering University College London London WC1E 7JE UK; ^4^ School of Chemical Sciences The University of Auckland Auckland 1010 New Zealand; ^5^ Key Laboratory of Comprehensive and Highly Efficient Utilization of Salt Lake Resources Qinghai Province Key Laboratory of Resources and Chemistry of Salt Lakes Qinghai Institute of Salt Lakes Chinese Academy of Sciences Xining Qinghai 810008 China; ^6^ Department of Materials Science and Metallurgy University of Cambridge Cambridge CB3 0FS UK

**Keywords:** electrode dissolution, fluorinated interphase, structural design, vanadium oxide, zinc‐ion batteries

## Abstract

Aqueous zinc‐ion batteries (AZIBs) have experienced a rapid surge in popularity, as evident from the extensive research with over 30 000 articles published in the past 5 years. Previous studies on AZIBs have showcased impressive long‐cycle stability at high current densities, achieving thousands or tens of thousands of cycles. However, the practical stability of AZIBs at low current densities (<1C) is restricted to merely 50–100 cycles due to intensified cathode dissolution. This genuine limitation poses a considerable challenge to their transition from the laboratory to the industry. In this study, leveraging density functional theory (DFT) calculations, an artificial interphase that achieves both hydrophobicity and restriction of the outward penetration of dissolved vanadium cations, thereby shifting the reaction equilibrium and suppressing the vanadium dissolution following Le Chatelier's principle, is described. The approach has resulted in one of the best cycling stabilities to date, with no noticeable capacity fading after more than 200 cycles (≈720 h) at 200 mA g^−1^ (0.47C). These findings represent a significant advance in the design of ultrastable cathodes for aqueous batteries and accelerate the industrialization of aqueous zinc‐ion batteries.

## Graphical abstract

Cathode dissolution significantly limits the cycle life of aqueous zinc‐ion batteries, particularly at low currents. An artificial constructed on V_6_O_13_ cathodes (ZnOTf‐LDH) is revealed to repel water, impede vanadium shuttling, and facilitate zinc transport. This suppresses vanadium dissolution and enables stable cycling for over 200 cycles at 200 mA g^−1^. The method is applicable to different vanadium oxide cathodes.

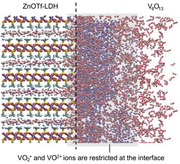

## 1 Introduction

**Figure 1:**
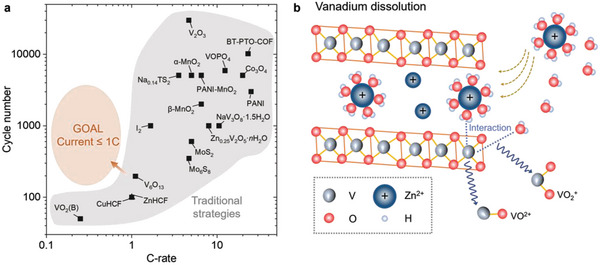
Current evaluation of cathodes for AZIBs and the schematic of V electrode dissolution. a) The published cathode data was analyzed in terms of C‐rate and cycle number. C‐rate values are either provided in the literature or calculated based on the highest reported discharge capacity and mass loading of active materials, respectively. The listed references include VO_2_(B),^[^ ^11^ ^]^ CuHCF,^[^ ^12^ ^]^ ZnHCF,^[^ ^13^ ^]^ V_6_O_13_,^[^ ^14^ ^]^ Mo_6_S_8_,^[^ ^15^ ^]^ MoS_2_,^[^ ^16^ ^]^ I_2_,^[^ ^17^ ^]^ Zn_0.25_V_2_O_5_·*n*H_2_O,^[^ ^1g^ ^]^ NaV_3_O_8_·1.5H_2_O,^[^ ^18^ ^]^ β‐MnO_2_,^[^ ^19^ ^]^ PANI,^[^ ^20^ ^]^ Na_0.14_TS_2_,^[^ ^21^ ^]^ α‐MnO_2_,^[^ ^1f^ ^]^ PANI‐MnO_2_,^[^ ^22^ ^]^ VOPO_4_,^[^ ^23^ ^]^ Co_3_O_4_,^[^ ^24^ ^]^ BT‐PTO‐COF,^[^ ^25^ ^]^ V_2_O_3_.^[^ ^26^ ^]^ The best performance in this work is also presented. b) Dissolution of vanadium oxide cathodes in AZIBs, the VO^2+^ and VO_2_^+^ are typically dissolved species.

Aqueous Zn‐ion batteries (AZIBs) promise inherent safety, environmental friendliness, high specific capacity, low cost, and fast charging.^[^ ^1^ ^]^ They hold great potential in replacing lithium‐ion batteries (LIBs), lead‐acid, and nickel‐metal hybrid batteries in wearable electronics and large‐scale energy storage.^[^ ^2^ ^]^ In the past decade, various strategies have been proposed in AZIBs to achieve energy and power densities that far exceed those of LIBs and seemingly superior cycling stability at high currents.^[^ ^3^ ^]^ Under such prosperity, many challenges underlying these claims of outstanding electrochemical performances are overlooked or underestimated. One critical issue is the poor capacity retention at low current densities (<1C, as illustrated in **Figure** **1**a) because small currents aggravate side reactions, such as the inevitable dissolution ofthe positive electrodes^[^ ^4^ ^]^ (Figure 1b, dissolution of vanadium oxides is selected as an example).

In previous research, the dissolution of electrode materials caused by H_2_O can be effectively alleviated by reducing H_2_O activity,^[^ ^5^ ^]^ such as water‐in‐salt^[^ ^6^ ^]^ and molecular crowding electrolytes.^[^ ^7^ ^]^ However, these strategies inevitably dissipate the unique advantages of aqueous electrolytes, such as high ionic conductivity, nonflammability, low cost, and nontoxicity. In common aqueous electrolytes where water activity is not attenuated, it is hard to desolvate Zn^2+^ due to its high hydration enthalpy (2046 kJ mol^−1^, as a comparison, the value of Li^+^ is 519 kJ mol^−1^)^[^ ^8^ ^]^ which seems to exacerbate the V‐H_2_O contact and derived V dissolution. On the other hand, the incomplete desolvation of Zn^2+^ brings the co‐intercalation of H_2_O and Zn^2+^ (or hydrated Zn^2+^).^[^ ^9^ ^]^ This co‐intercalation was previously considered a process that stabilizes the lattice.^[^ ^10^ ^]^

On the contrary, our DFT simulations found that the intercalated hydrated Zn^2+^ generates large cavities within the lattice spacing; under such circumstances, the co‐intercalated free H_2_O coordinates with the partially coordinated V accelerates the V dissolution. We thus introduced a zinc‐permeable, hydrophobic, and vanadium‐impermeable artificial interphase on the vanadium oxide (VO*_x_*) cathodes. Under our hypotheses, such an artificial interphase could radically inhibit the intercalation of H_2_O, and substantially mitigates V dissolution through adjusting local reaction equilibrium. Our strategy turns out to be so effective that the full cell achieves one of the best cycling stabilities under low current densities (<1 C) to date (almost no capacity fades after 200 cycles). Considering the clear understanding of the capacity fading mechanism and the target design of the composite cathode structures, this work provides a universal design strategy for the construction of highly stable AZIBs.

## 2 Results and Discussion

In this study, V_6_O_13_ possessing alternating single and double VO*_x_* layers was selected as a model cathode material, which can be considered as a mixed V_2_O_5_ (single layer) and VO_2_ structure (double‐layered),^[^ ^1c^ ^]^ exhibiting the representative structures of vanadium oxide cathodes. The morphology of the material comprises stacked nanoribbons, as observed in the scanning electron microscope (SEM) image (Figure S1a, Supporting Information). This morphology provides a larger specific surface area and shorter ion diffusion path than bulk ones, enabling a complete reaction and aiding our understanding of the reaction process. The powder X‐ray diffraction (XRD) pattern shown in Figure S1b (Supporting Information) confirms the successful synthesis of pure phase V_6_O_13_ material (JCPDS No. 19‐1399). Additionally, our X‐ray photoelectron spectroscopy (XPS) analysis of the V 2p region (Figure S1c, Supporting Information) reveals the mixed valence states of V. The Raman spectrum presented in Figure S2 (Supporting Information) further confirms the material's identity as V_6_O_13_. Collectively, these characterizations provide basic structural information of the synthesized material.

**Figure 2:**
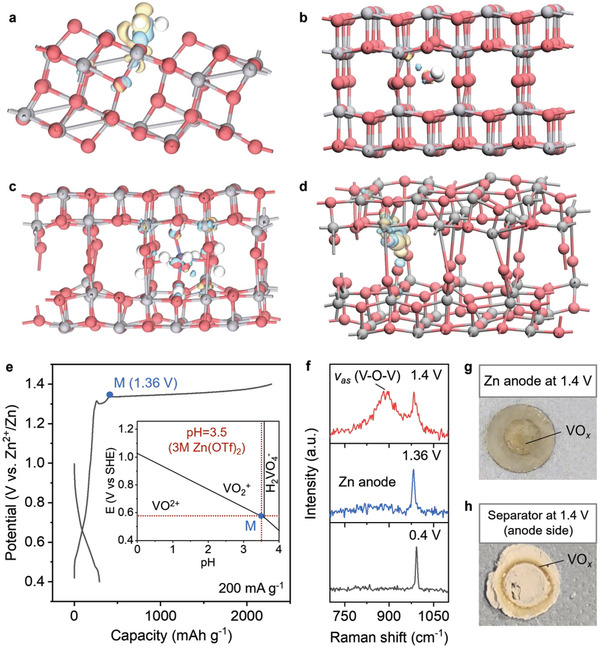
Theoretical and experimental investigations of the V_6_O_13_ dissolution in H_2_O. a–d) The charge density difference plots from density functional theory (DFT) calculations (the isovalue was set as 0.05, gray, red, and white balls represent V, O, and H atoms, respectively, and yellow and blue surfaces correspond to the loss and gain of charge, respectively), including between free H_2_O and original surface exposing (011) plane of V_6_O_13_ a), between free H_2_O and original bulk V_6_O_13_ b), between H_2_O from the intercalated hydrated Zn^2+^ and V_6_O_13_ c), and between free H_2_O and V_6_O_13_ after hydrated Zn^2+^ intercalation d). e) Galvanostatic charge/discharge curve of Zn||V_6_O_13_ cell at a current density of 200 mA g^−1^. The inset is the amplified V‐H_2_O Pourbaix diagram, while the whole diagram is shown in Figure S3, Supporting Information. The red dotted line indicates a pH of 3.5, which is equivalent to the pH of 3 m Zn(OTf)_2_. The voltage at this pH is 1.36 V versus Zn/Zn^2+^ and is attributed to a boundary between VO^2+^ and VO_2_^+^. f) Raman spectrum of the Zn foil anode at different charge/discharge states during the first cycle. g,h) Optical images of the Zn foil anode g) and the anode‐side separator h) at 1.4 V during the first charge process.

We conducted a series of simulations about the interaction between free H_2_O and V_6_O_13_ under different circumstances to understand the dissolution behavior of V_6_O_13_ (−0.07 eV A^−2^, Table S1, Supporting Information). **Figure** **2**a shows a certain charge transfer between the free H_2_O and the V_6_O_13_ outer surface, demonstrating the strong interaction between the fluent H_2_O and the uncoordinated V atom due to the broken periodicity. Such interaction leads to the dissolution phenomenon of VO*_x_* materials in H_2_O, as suggested by previous research.^[^ ^27^ ^]^ Next, we examined the interaction between the free H_2_O and the fully coordinated V_6_O_13_ lattice. As shown in Figure 2b, the interaction of H_2_O did not break the full coordination environment of V atoms, which results in an even weaker interaction than the external VO*_x_* surface. However, a relatively small distortion of the VO*_x_* lattice has caught our attention tthat the bulk structure of VO*_x_* is not as stable as we thought. Even such a small perturbation can cause deformation of the structure, let alone the hydrated Zn^2+^ that is often observed in AZIBs (Figure 2c). Notable deformation surges as we expected while hardly transferred electrons were observed, indicating the tight binding between the H_2_O and Zn^2+^ in Zn(H_2_O)_6_^2+^. Such a finding is in full consistency with the large hydration enthalpy of Zn^2+^ (2046 kJ mol^−1^, Table S2, Supporting Information). This in turn explains why the intercalation of hydrated Zn^2+^ occurs, since Zn^2+^ is difficult to dehydrate.^[^ ^28^ ^]^

Experimentally, the dissolution progress basically follows the reaction equation below, which describes the reaction between V_2_O_5_ (the ideal charging product of VO*_x_* cathode) and H_2_O under acidic conditions

(1) $$1/2V_{2}O_{5(s)}+H^{+}=VO_{2}^{+}+1/2H_{2}O$$

Referring to the V‐H_2_O Pourbaix diagram (inset in Figure 2e), we have determined that the transition from VO^2+^ to VO_2_^+^ occurs at ≈1.36 V. Our experiments have also shown a prolonged charging plateau during the first charging progress, with the turning point near 1.36 V (Figure 2e). It is speculated that the VO*_x_* solid dissolves continuously during this charging process, generating VO_2_^+^. Intriguingly, the dissolved VO_2_^+^ migrates to the anode under the charging electric field. This is supported by the detection of a VO*_x_* signal on the Zn anode surface at the end of the charge (Figure 2f), as well as the observation of an earthy yellow precipitate on the Zn anode and on the anode‐side separator (Figure 2g,h). Electrochemical impedance spectroscopy (EIS) measurements further indicate an additional interface emerges at the end of the long charging plateau, likely corresponding to the deposition of dissolved V species and the subsequent generation of a VO*_x_* precipitation layer on the Zn anode surface (Figure S4, Supporting Information).

However, the intercalation of Zn(H_2_O)_6_^2+^ leads to a pronounced lattice distortion, allowing the appearance of many suspended bonds, like the state of the exposed VO*_x_* surface in Figure 2a. Therefore, we considered the interaction between the free H_2_O in the aqueous electrolyte and the lattice when hydrated Zn^2+^ is intercalated, as shown in Figure 2d. As expected, an even greater degree of charge was transferred between the broken lattice with free H_2_O than that on the surface of the V_6_O_13_. This state is anticipated to display a greater degree of V dissolution due to the similarly exposed unsaturated surfaces of both the upper and lower VO*_x_* layers, which is fully consistent with the experiments. Based on this theoretical finding, we assembled the Zn||V_6_O_13_ full cell for further study. To prevent V dissolution in the VO*_x_* cathode, it is necessary to modify the interaction between H_2_O and the electrode. To address this issue, we developed a cathode‐electrolyte interlayer (CEI) that could effectively manage this situation. Previous studies reported a CEI of Zn*_x_* (OTf)*_y_* (OH)_2_ *_x_*_−_ *_y_* ·*n*H_2_O (ZnOTf‐LDH) that could be generated in situ on the VO*_x_* cathode due to the irreversible intercalation of H^+^. Interestingly, the generation of such ZnOTf‐LDH did not immediately result in the capacity fading. Inspired by this phenomenon, we carefully investigated its species permeability.

**Figure 3:**
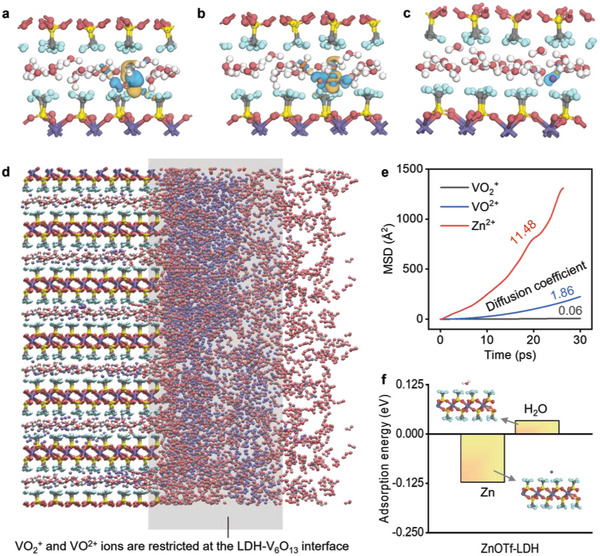
Optimized structure of ZnOTf‐LDH and corresponding interactions of VO^2+^, VO_2_^+^, Zn^2+^, and H_2_O within it or across it. a–c) The charge density difference when VO^2+^ a), VO_2_^+^ b), and Zn^2+^ c) transfer in the ZnOTf‐LDH individually. the Isovalue was set as 0.03. d) Scheme of the confinement effect of ZnOTf‐LDH on VO^2+^ and VO_2_^+^. e) The mean square displacement of different ions with LDH. f) Adsorption energy of Zn^2+^ and H_2_O in the channel of ZnOTf‐LDH.

Through DFT calculations and differential charge density analysis, we found that VO^2+^ and VO_2_^+^ in the channel undergo severe charge transfer with ZnOTf‐LDH, while Zn^2+^ does not (**Figure** **3**a–c). This indicates that VO^2+^ or VO_2_^+^ can be trapped in the H_2_O network in the ZnOTf‐LDH, while Zn^2+^ could pass through the LDH easily. Therefore, ZnOTf‐LDH could confine VO^2+^ and VO_2_^+^ at the interface between ZnOTf‐LDH and V_6_O_13_ (Figure 3d), preventing further V dissolution. This was verified using ab initio molecular dynamics (AIMD), which showed strong Zn^2+^ permeability but weak permeability of VO^2+^ and VO_2_^+^ (Figure 3e). Additionally, ZnOTf‐LDH was proved to be hydrophobic and highly Zn affinitive (Figure 3f). Such quality could desolvate Zn(H_2_O)_6_^2+^ and prevent the secondary H_2_O intercalation phenomenon found in Figure 2d.

**Figure 4:**
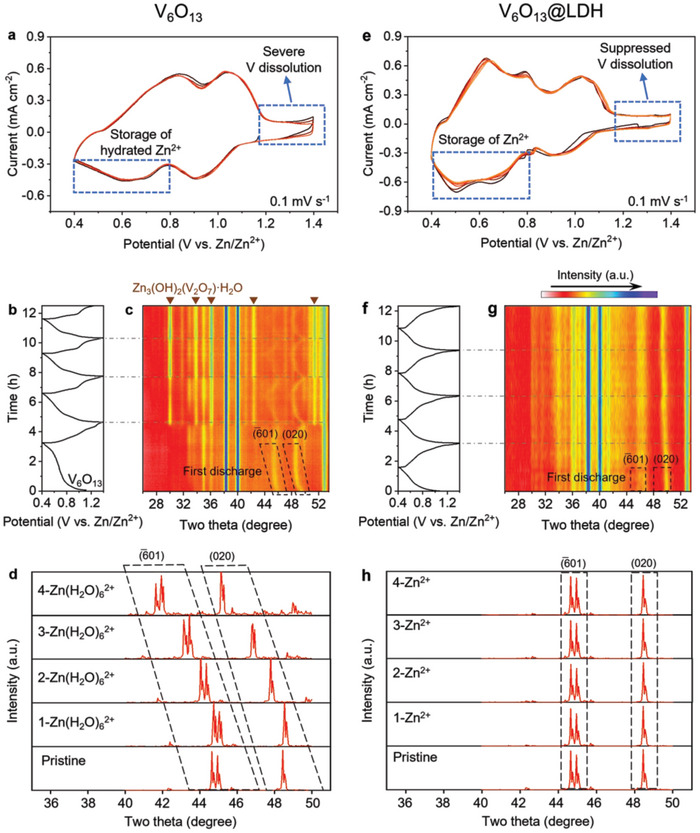
Intercalation behavior within V_6_O_13_ and V_6_O_13_@LDH. a–d) Electrochemical measurements and characterizations of V_6_O_13_ including CV curves with a scan rate of 0.1 mV s^−1^ a), in situ XRD characterizations and corresponding charge/discharge curves b,c), and simulated shifts of the diffraction peaks during the 1st discharging d). The JCPDS No. of the Zn_3_(OH)_2_(V_2_O_7_)·H_2_O shown in (c) is 01‐087‐0417. e–h) Electrochemical measurements and characterizations of V_6_O_13_@LDH including CV curves with a scan rate of 0.1 mV s^−1^ e), in situ XRD characterizations and corresponding charge/discharge curves f,g), and simulated shifts of the diffraction peaks during the 1st discharging h). The $\bar{(6}$01) and (020) crystal planes of V_6_O_13_ are labeled in (c,d,g,h).

In addition, we examined the intercalation behavior of Zn^2+^ and hydrated Zn^2+^ in V_6_O_13_ through DFT calculations (Figure S5, Supporting Information). For Zn^2+^, there are two diffusion channels (paths 1 and 2) with corresponding energy barriers of 0.53 and 0.68 eV, while hydrated Zn^2+^ has only one diffusion channel (path 1) with a higher energy barrier of 1.54 eV due to the size effect. The result indicates that the introduction of ZnOTf‐LDH may facilitate subsequent diffusion of Zn^2+^ in the bulk phase after its desolvation. Previous literature reports that ZnOTf‐LDH has a shedding problem due to H^+^ deintercalation, which dissolves the ZnOTf‐LDH at the interface. To address this issue, we chemically synthesized ZnOTf‐LDH and stuck it to the V_6_O_13_ cathode surface (noted as V_6_O_13_@LDH). The cyclic voltammetry (CV) measurements (**Figure** **4**a,e) indicate that the curves of V_6_O_13_ are discrete in the first few cycles near 1.4 V, while those of V_6_O_13_@LDH are overlapping, indicating V dissolution is suppressed after introducing the ZnOTf‐LDH CEI. Interestingly, at the Zn^2+^ intercalation potential around 0.6 V, V_6_O_13_ shows only one broadened CV peak, while V_6_O_13_@LDH has two separated CV peaks at 0.503 and 0.656 V, corresponding to the intercalation of hydrated Zn^2+^ and Zn^2+^ as shown in the theoretical simulation, respectively (Figure S5, Supporting Information). Moreover, we carried out variable temperature EIS tests. The charge transfer activation energy (*E*_a_) can be determined through plotting the ln(*R*_ct_^−1^) and ln(σ*_w_* ^−1^) versus reciprocal temperature (Figure S6, Supporting Information), which obey the Arrhenius equation

(2) $$1/R_{\mathrm{ct}}^{-1}=\;A\exp\left(-E_{a1}/RT\right)$$

(3) $$1/\sigma_{w}^{-1}=A\exp\left(-E_{a2}/RT\right)$$

where *A* is the frequency factor, *E*_a1_ is the activation energy for interfacial charge transfer, *E*_a2_ is the activation energy for solid‐state charge transfer, *R* is the gas constant, and *T* is absolute temperature. The V_6_O_13_@LDH exhibits a higher *E*_a1_ and lower *E*_a2_ than V_6_O_13_, indicating an increased interfacial resistance due to the introduction of a CEI and a decreased solid‐state diffusion resistance. The decreased solid‐state ionic diffusion can be attributed to the intercalated ions transitioning from hydrated Zn^2+^ to Zn^2+^. We further performed in situ XRD tests on both samples. Regarding the V_6_O_13_ (Figure 4b,c), the XRD peaks corresponding to the two crystal planes ($\bar{6}$01) (initially at 46°) and (020) (initially at 49.5°) are shifted to lower angles during discharging and to higher angles during charging, corresponding to the increase and then decrease of the interlayer spacing,^[^ ^11^ ^]^ respectively, while the peak positions in V_6_O_13_@LDH exhibit no significant shift (Figure 4f,g). This suggests (de)intercalation of the large‐size hydrated Zn^2+^ in V_6_O_13_, while Zn^2+^ in V_6_O_13_@LDH. In this regard, we performed simulations toward XRD using DFT calculations based on the (de)intercalated hydrated Zn^2+^ and Zn^2+^ in the bulk phase of V_6_O_13_, respectively (Figure 4d,h), and found that the intercalation of hydrated Zn^2+^ did cause a significant increase in the interlayer spacing, while Zn^2+^ induces negligible increase, which is consistent with the in situ XRD results. These results suggest that ZnOTf‐LDH can function as described in Figure 3.

**Figure 5:**
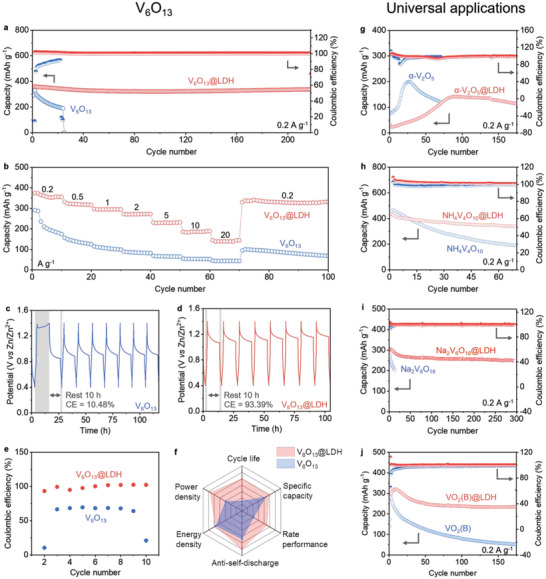
Comprehensive performance and universal applications of the artificial CEI of ZnOTf‐LDH. a) Cycling performance of V_6_O_13_ and V_6_O_13_@LDH at a current density of 0.2 A g^−1^. b) Rate performance of V_6_O_13_ and V_6_O_13_@LDH at varying current densities. c–e) Self‐discharge performances of V_6_O_13_ and V_6_O_13_@LDH. The current density is 0.2 A g^−1^. e) is the statistics of c) and d). f) Radar chart comparing cycle life, specific capacity, rate performance, antiself‐discharge, energy density, and power density of V_6_O_13_ and V_6_O_13_@LDH. g–j) Cycling performance of α‐V_2_O_5_ and α‐V_2_O_5_@LDH g), NH_4_V_4_O_10_ and NH_4_V_4_O_10_@LDH h), Na_2_V_6_O_16_ and Na_2_V_6_O_16_@LDH i), and VO_2_(B) and VO_2_(B)@LDH j) at 0.2 A g^−1^.

Furthermore, we tested the electrochemical performance of the cathodes with and without ZnOTf‐LDH coatings. At a low current density of 200 mA g^−1^, the capacity retention of V_6_O_13_@LDH presented 98.3% after over 200 cycles (i.e., ≈720 h, as seen in **Figure** **5**a), which is the best cycling performance at low current densities so far (Figure 1a and **Table** **1**). The Coulombic efficiency values of V_6_O_13_ during initial cycles were over 100%, corresponding to the severe V dissolution and shuttling of V species. The improved cycling performance is inextricably linked to our proposed mechanism, which is based on Le Chatelier's principle, i.e., elevating local VO^2+^ concentration and decreasing H_2_O concentration through the function of ZnOTf‐LDH, thus shifting the reaction equilibrium in Equation (1) to the left and inhibiting further V dissolution.^[^ ^29^ ^]^ In addition, the rate performance of V_6_O_13_@LDH is also much higher than that of V_6_O_13_ (Figure 5b), due to the intercalation of Zn^2+^ in the former and hydrated Zn^2+^ in the latter. As seen in Figure 5c–e, the capacity retention of V_6_O_13_@LDH after resting is consistently much higher than that of V_6_O_13_, indicating that the ZnOTf‐LDH suppresses the spontaneous ion exchange at the interface and thus obtaining a stable interface. Ultimately, the cycle life, specific capacity, rate performance, antiself‐discharge, energy density, and power density of V_6_O_13_@LDH are all much more improved than those of V_6_O_13_ (Figure 5f). In the end, we applied this ZnOTf‐LDH approach to other typical VO*_x_* phases, including α‐V_2_O_5_, NH_4_V_4_O_10_, Na_2_V_6_O_10_, and VO_2_(B) (Figure 5g–j). Our results demonstrate a significant improvement in cycling performance at a current density of 200 mA g^−1^ across various VO*_x_* cathode materials, highlighting the generalizability of our strategy.

**Table 1:** Performance comparison

Samples	Capacity retention	Current density	Electrolyte	Refs.
Zn_0.25_V_2_O_5_·*n*H_2_O	89.9% after 50 cycles	300 mA g^−1^	1 m ZnSO_4_	[1g]
VO*_x_*‐rGO	95.8% after 50 cycles	100 mA g^−1^	3 m Zn(OTf)_2_	[30]
Oxygen‐deficient V_6_O_13_	96.7% after 50 cycles	200 mA g^−1^	3 m Zn(OTf)_2_	[31]
Zn_0.52_V_2_O_5‐_ *_a_* ·1.8H_2_O	97.5% after 50 cycles	200 mA g^−1^	3 m Zn(OTf)_2_	[32]
PEDOT‐NH_4_V_3_O_8_·0.5H_2_O	94.8% after 100 cycles	200 mA g^−1^	3 m Zn(OTf)_2_	[33]
HfO_2_‐coated Zn_3_V_2_O_7_(OH)_2_·2H_2_O	90% after 100 cycles	100 mA g^−1^	1 m ZnSO_4_	[34]
V_2_O_3_@C	92.1% after 150 cycles	200 mA g^−1^	3 m Zn(OTf)_2_	[35]
Zn_0.1_V_2_O_5_·*n*H_2_O	93% after 200 cycles	200 mA g^−1^	2 m ZnSO_4_	[36]
V_6_O_13_	80% after 200 cycles	400 mA g^−1^	3 m Zn(OTf)_2_	[14]
V_6_O_13_@ZnOTf‐LDH	98.3% after 218 cycles	200 mA g^−1^	3 m Zn(OTf)_2_	This work

## 3 Conclusion

In conclusion, this study has identified that hydrated Zn^2+^ intercalation triggers intercalation of free H_2_O, which intrinsically leads to severe V dissolution in VO*_x_* cathodes. To address this issue, we designed and synthesized ZnOTf‐LDH as an artificial CEI on the V_6_O_13_ surface, which repels H_2_O, blocks VO^2+^, and VO_2_^+^, and allows the transport of Zn^2+^. This reduces the reactant H_2_O and confines the generated VO^2+^ and VO_2_^+^, thus effectively suppressing the V dissolution following Le Chatelier principle. Therefore, we achieved stable cycling performance (over 200 cycles without remarkable capacity fading) at a low current density (200 mA g^−1^). Implementing detailed characterizations, including in situ XRD, DFT calculations, and electrochemical analysis, the CEI also enabled a higher Zn^2+^ diffusion kinetics originating from facilitated solid‐state diffusion (Zn^2+^ rather than Zn(H_2_O)_6_^2+^) and desolvation of hydrated Zn^2+^. This strategy was universally applied to α‐V_2_O_5_, NH_4_V_4_O_10_, Na_2_V_6_O_10_, and VO_2_(B) systems, significantly improving their cycling stability at low current densities. Our findings offer new insights into the dissolution problem of V‐based materials and provide a pervasive strategy to promote practical high‐capacity AZIBs.

## 4 Experimental Section

### Synthesis of V_6_O_13_

In the typical synthesis process, 1.2 g of V_2_O_5_ and 1.8 g of H_2_C_2_O_4_⋅2H_2_O were added to 40 mL of deionized (DI) water. The resulting mixture was then magnetically stirred at 75 °C for 1 h, yielding a dark blue solution. Subsequently, this solution was transferred into a 50 mL Teflon‐lined autoclave and kept at 180 °C for 3 h. After the completion of the reaction, the autoclave was allowed to cool down to room temperature naturally. The resulting products were washed with ethanol and DI water, then dried at 60 °C overnight before being collected.

### Synthesis of Zn*_x_* (OTf)*_y_* (OH)_2_ *_x_*_–_ *_y_* ·nH_2_O

The synthesis of Zn*_x_* (OTf)*_y_* (OH)_2_ *_x_*_–_ *_y_* ·*n*H_2_O was adapted from two previous papers.^[^ ^37^ ^]^ Specifically, 1 mL of 1 m KOH was added dropwise into 9 mL of 3 m Zn(CF_3_SO_3_)_2_ solution. The resulting white precipitate was then collected after centrifugation at 4400 rpm for 10 min. The supernatant liquid was decanted, and the resulting white precipitate was washed with water and dried for ≈30 min using a rotary evaporator equipped with a water bath held at 60 °C.

### Synthesis of Universal Samples

The α‐V_2_O_5_ was used as purchased, while the synthesis methods for NH_4_V_4_O_10_,^[^ ^38^ ^]^ Na_2_V_6_O_16_,^[^ ^39^ ^]^ and VO_2_(B)^[^ ^11^ ^]^ were directly adapted from previous literature.

### Materials Characterizations

XRD and in situ XRD measurements were conducted using a Bruker D8 Discover X‐ray diffractometer with an area detector using Cu Kα X‐ray source. XPS analysis was performed with a VG Multilab 2000 instrument, while the Raman spectra were acquired using a Horiba LabRAM HR Evolution with an excitation laser of 532 nm. Field‐emission SEM images were obtained on a JEOL‐JSM‐6700F with a voltage of 5 kV and an emission current of 110.8 µA.

### Electrochemical Measurements

The working cathode of V_6_O_13_ comprised active materials (70 wt%), acetylene black conductive additive (20 wt%), and polyvinylidene fluoride (PVDF) (10 wt%). In contrast, the working cathode of V_6_O_13_@LDH contained active materials (60 wt%), Zn*_x_* (OTf)*_y_* (OH)_2_ *_x_*_–_ *_y_* ·*n*H_2_O (10 wt%), acetylene black conductive additive (20 wt%), and polyvinylidene fluoride (PVDF) (10 wt%). After grinding for 60 min, an appropriate amount of NMP was added to form a homogenous ink, and then it was cast onto carbon paper and dried at 60 °C overnight. Subsequently, the carbon paper was punched into circular sheets with a diameter of 16 mm to serve as cathodes. 2032 coin cells were assembled with the cathode, glass fiber filter paper as a separator, zinc metal foil as an anode. The electrolyte used was 3 m Zn(CF_3_SO_3_)_2_ aqueous solution. Galvanostatic charge/discharge tests were undertaken on a Neware battery test system (Shenzhen, China). Additionally, CV and EIS were performed based on Swagelok cells, using a Biologic VMP‐3 electrochemical workstation.

### Computational Methods and Models

All the DFT simulations in the work were performed within a periodic model by the Vienna ab initio simulation program (VASP).^[^ ^40^ ^]^ The generalized gradient approximation (GGA) was used with the Perdew–Burke–Ernzerhof (PBE) exchange‐correlation functional.^[^ ^41^ ^]^ The projector‐augmented wave (PAW) method^[^ ^42^ ^]^ was utilized to describe the interactions of the electron‐nucleus, and the cut‐off energy for the plane‐wave basis set was 450 eV. Brillouin zone integration was set as 2×2×1 Monkhorst–Pack *k*‐point mesh for systems with surfaces. All the adsorption geometries were optimized using a force‐based conjugate gradient algorithm. The adsorption energy (Δ*G*_ad_) was defined as

(4) $$\Delta E_{\mathrm{ad}}\;=\;E_{\mathrm{adsorbate}+\mathrm{surface}-}E_{\mathrm{adsorbate}}-E_{\mathrm{surface}}$$

where *E*_surface_, *E*_adsorbate_, and *E*_adsorbate+surface_ are the free energies of the surface, adsorbate in the gas phase, and adsorbate adsorbed on the surface, respectively. The climbing image nudged elastic band (CINEB) method^[^ ^43^ ^]^ was applied to find the transition state for the migration barrier. Moreover, the criteria of energy and force convergence were set to 1.0 × 10^−5^ eV per atom and 0.02 eV Å^−1^ for geometry optimization, respectively. AIMD was used in this work using the CASTEP module, and the time step was 0.5 fs for 30 ps to obtain the MSD (mean square distance). The temperature of the MD simulation was oscillating controlled using the algorithm of Nose–Hoover thermostats,^[^ ^44^ ^]^ and the average temperature was set to 300 K.

## Conflict of Interest

The authors declare no conflict of interest.

## Supporting information

Supporting Information

## Data Availability

The data that support the findings of this study are available from the corresponding author upon reasonable request.
