# Supplementary material for: Inhibition of Vanadium Cathodes Dissolution in Aqueous Zn‐Ion Batteries
Source: Adv Mater. 2024 Jan 23;36(14):2310645. doi: 10.1002/adma.202310645 (PMC11475447; doi:10.1002/adma.202310645)
Supplement: Supplementary file 1 — Supporting Information [file ADMA-36-2310645-s001.pdf]

ADVANCED MATERIALS

Supporting Information

for *Adv. Mater.*, DOI 10.1002/adma.202310645

Inhibition of Vanadium Cathodes Dissolution in Aqueous Zn-Ion Batteries

Yuhang Dai, Chengyi Zhang, Jianwei Li, Xuan Gao, Ping Hu, Chumei Ye, Hongzhen He, Jiexin Zhu, Wei Zhang, Ruwei Chen, Wei Zong, Fei Guo, Ivan P. Parkin, Dan J. L. Brett, Paul R. Shearing, Liqiang Mai and Guanjie He**

Supporting Information

Inhibition of vanadium cathodes dissolution in aqueous Zn-ion batteries

Yuhang Dai[1,3,4,7], Chengyi Zhang[2,6], Jianwei Li[5,6], Xuan Gao[1,6], Ping Hu[3], Chumei Ye[6], Hongzhen He[1,4], Jiexin Zhu[1,3], Wei Zhang[1], Ruwei Chen[1], Wei Zong[1], Fei Guo[1], Ivan P. Parkin[1], Dan J.L. Brett[4], Paul R. Shearing[4], Liqiang Mai[3,*], Guanjie He[1,*]

[1] Christopher Ingold Laboratory, Department of Chemistry, University College London, London WC1H 0AJ, UK

[2] School of Chemical Sciences, The University of Auckland, Auckland 1010, New Zealand

[3] State Key Laboratory of Advanced Technology for Materials Synthesis and Processing, Wuhan University of Technology, Wuhan 430070, China

[4] Electrochemical Innovation Lab, Department of Chemical Engineering, University College London, London WC1E 7JE, UK

[5] Key Laboratory of Comprehensive and Highly Efficient Utilization of Salt Lake Resources, Qinghai Province Key Laboratory of Resources and Chemistry of Salt Lakes, Qinghai Institute of Salt Lakes, Chinese Academy of Sciences, Xining, Qinghai 810008, P. R. China

[6] Department of Materials Science and Metallurgy, University of Cambridge, Cambridge CB3 0FS, UK

[7] These authors contributed equally to this work.

[*] Email: mlq518@whut.edu.cn; g.he@ucl.ac.uk

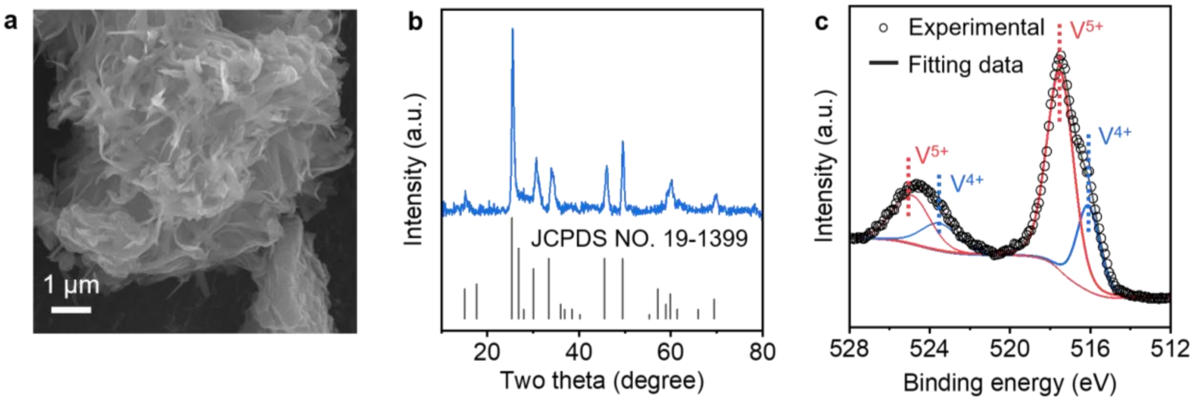

Figure S1. Structure of the synthesized V_6O_{13}. a) Scanning electron microscope (SEM) image. b) X-ray diffraction (XRD) pattern. c) X-ray photoelectron spectroscopy (XPS) spectrum of the V 2p core level in V_6O_{13}.

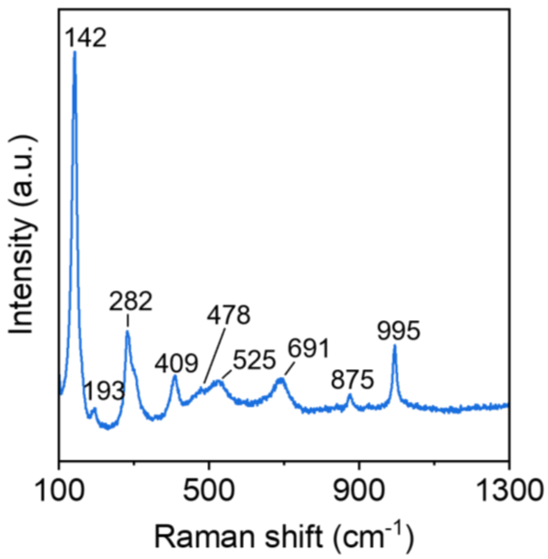

Figure S2. Raman spectrum of the synthesized V_6O_{13}.

Table S1. Surface energy values of different planes of V_6O_{13}.

Surface	(100)	(011)	$(10\bar{4})$	$(10\bar{6})$
Surface energy (eV Å^{-2})	0.02	-0.07	0.03	0.03

Table S2. Experimental hydration values of different cations[1].

**Ion**	**-Δ*h*$_{hydration}$ (kJ mol^{-1})**
H^+	1091
Li^+	519
Na^+	409
K^+	322
Rb^+	293
Cu^+	593
Ag^+	473
NH_4^+	307
Mg^{2+}	1921
Ca^{2+}	1577

Ba^{2+}	1305
Mn^{2+}	1841
Fe^{2+}	1946
Co^{2+}	1996
Ni^{2+}	2105
Cu^{2+}	2100
**Zn^{2+}**	**2046**
Al^{3+}	4665
Fe^{3+}	4430

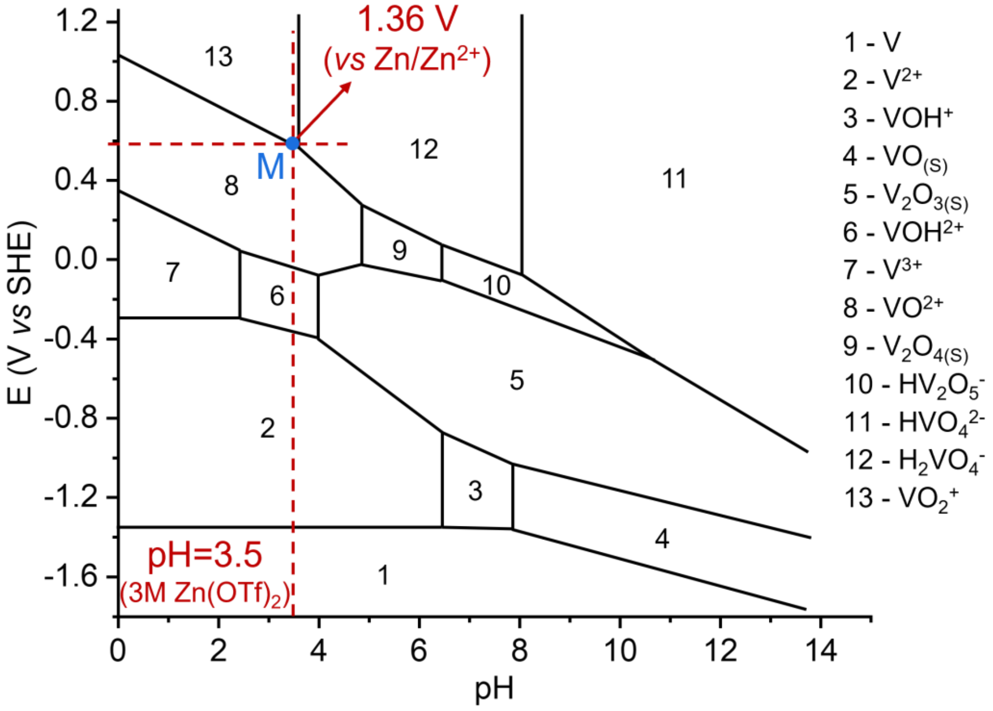

Figure S3. Modified V-H_2O Pourbaix diagram[2].

The red dotted line indicates a pH of 3.5, which is equivalent to the pH of 3 M $Zn(OTf)_2$. The voltage at this pH is of 1.36 V *vs.* Zn/Zn^{2+} and is attributed to a boundary between VO^{2+} and VO_2^+.

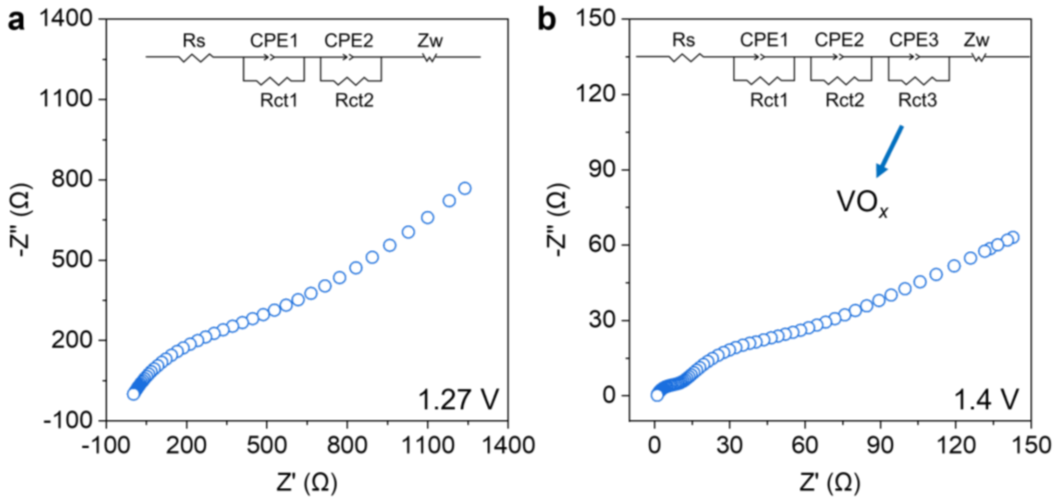

Figure S4. Electrochemical impedance spectroscopy (EIS) spectra of the Zn-V_6O_{13} cell at (a) 1.27 V and (b) 1.4 V *vs.* Zn/Zn^{2+} during the first charging process.

The newly formed R_{ct3}/CPE3 corresponds to the generation of VO_x on the Zn surface.

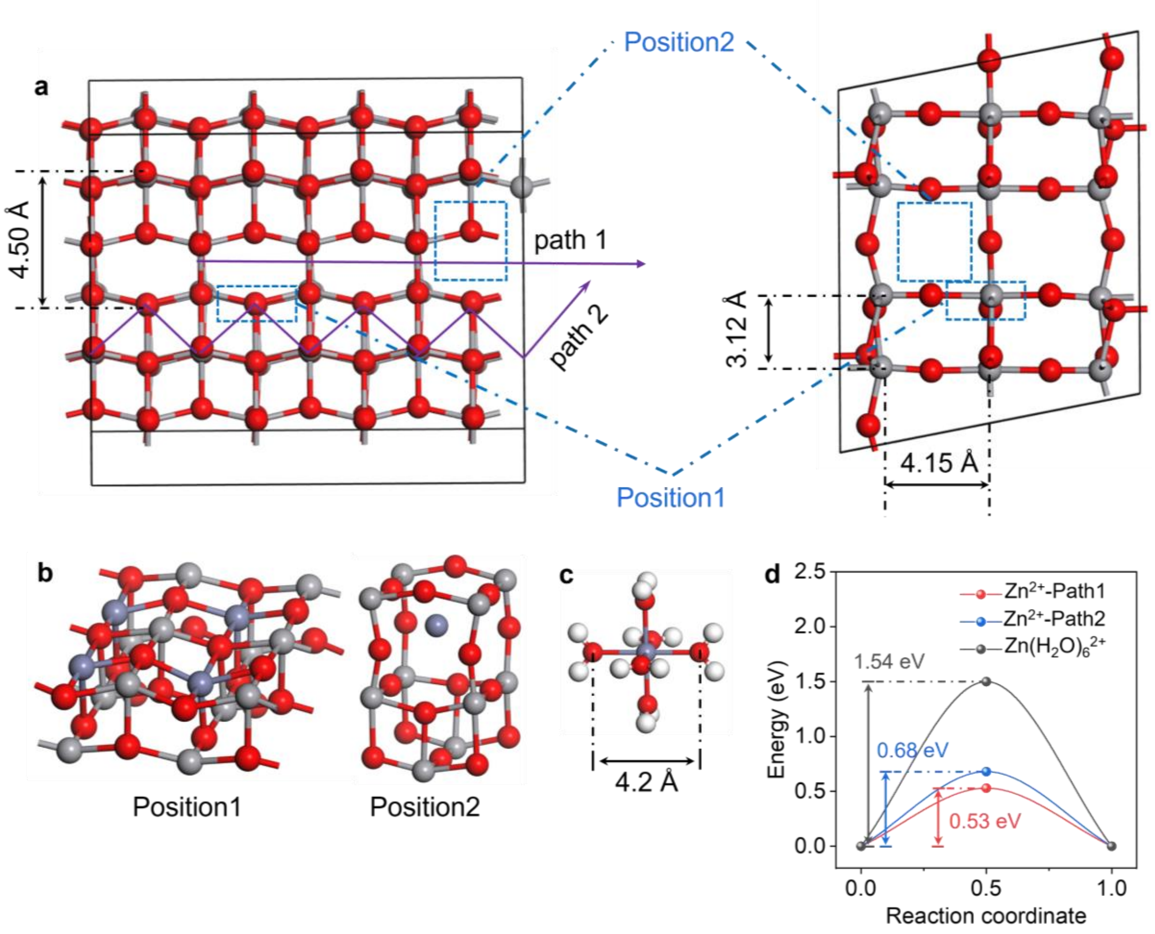

Figure S5. The migration paths and coordination sites of Zn^{2+} and $Zn(H_2O)_6^{2+}$ in the V_6O_{13} are examined. a) Two coordination sites and paths are identified for the migration of Zn, while only

one site and path are observed for $Zn(H_2O)_6^{2+}$ due to the size effect. b) A detailed analysis is presented regarding the positioning of Zn^{2+} in position 1 and position 2. c) The structure of $Zn(H_2O)_6^{2+}$ is described, noting that it remains unchanged when the Zn^{2+} fully occupies the sites. The main focus of this investigation is thus centered on the insertion of $Zn(H_2O)_6^{2+}$ and its impact on the structural changes. d) The transition barrier when Zn^{2+} or $Zn(H_2O)_6^{2+}$ migrates in the structure.

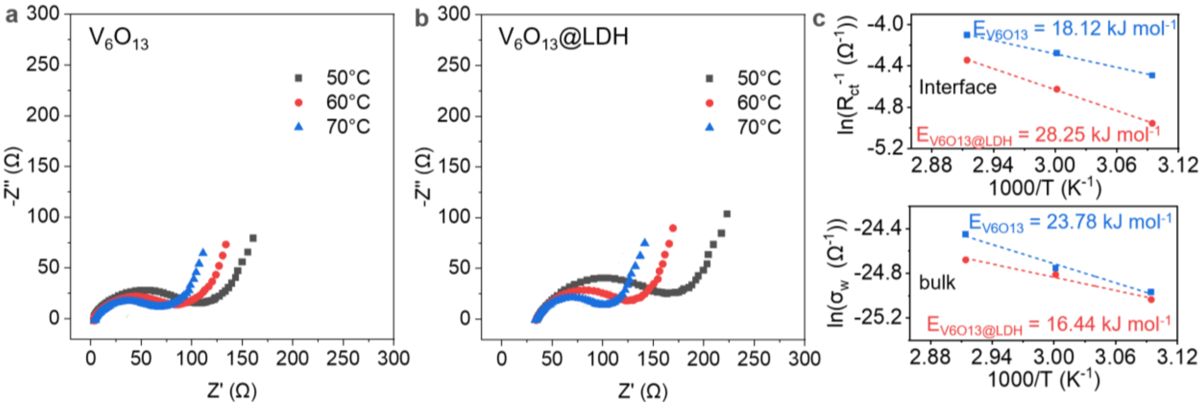

Figure S6. a,b) EIS spectra of V_6O_{13} (a) and V_6O_{13}@LDH (b) at viable temperatures. c) Arrhenius plots of R_{ct}^{-1} values at different temperatures (from 50 °C to 70 °C).

The activation energies for the interfacial charge transfer processes were determined by calculating the slope of the fitted line.

Supporting Notes

1. Homogeneity of the V_6O_{13}@LDH electrode.

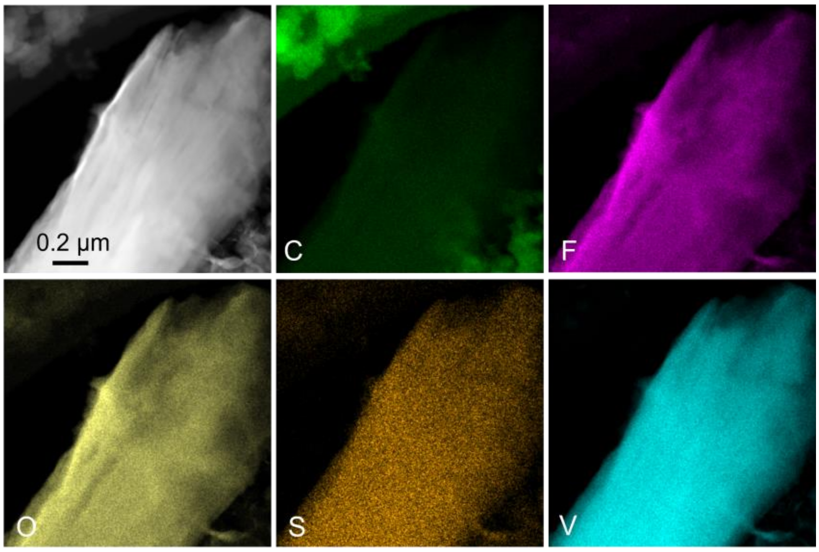

Figure S7. TEM image and corresponding EDS mappings of the pristine V_6O_{13}@LDH electrode.

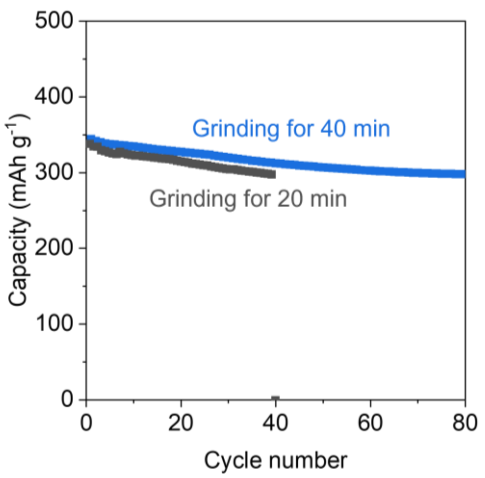

Figure S8. The galvanostatic cycling performance of the V_6O_{13}@LDH electrode that was ground for 20 min and 40 min, respectively, during the electrode preparation process.

As shown in Figure S7, C, F, O, S, and V elements are distributed uniformly in the electrode, indicating the homogeneous contact of ZnOTf-LDH with V_6O_{13} in the V_6O_{13}@LDH electrode, probably due to the long grinding time of up to 60 min. To investigate the effect of grinding time (slurry homogeneity) on the cycling performance, we also prepared the contrasting V_6O_{13}@LDH electrode by grinding for 20 min and 40 min, respectively. As depicted in Figure S8, the inefficient mixing process resulted in rapid capacity fading of the electrode, revealing that the inhibition of vanadium dissolution is compromised when ZnOTf-LDH is not sufficiently homogeneously mixed with V_6O_{13}.

2. Corroboration of DFT calculations and experiments

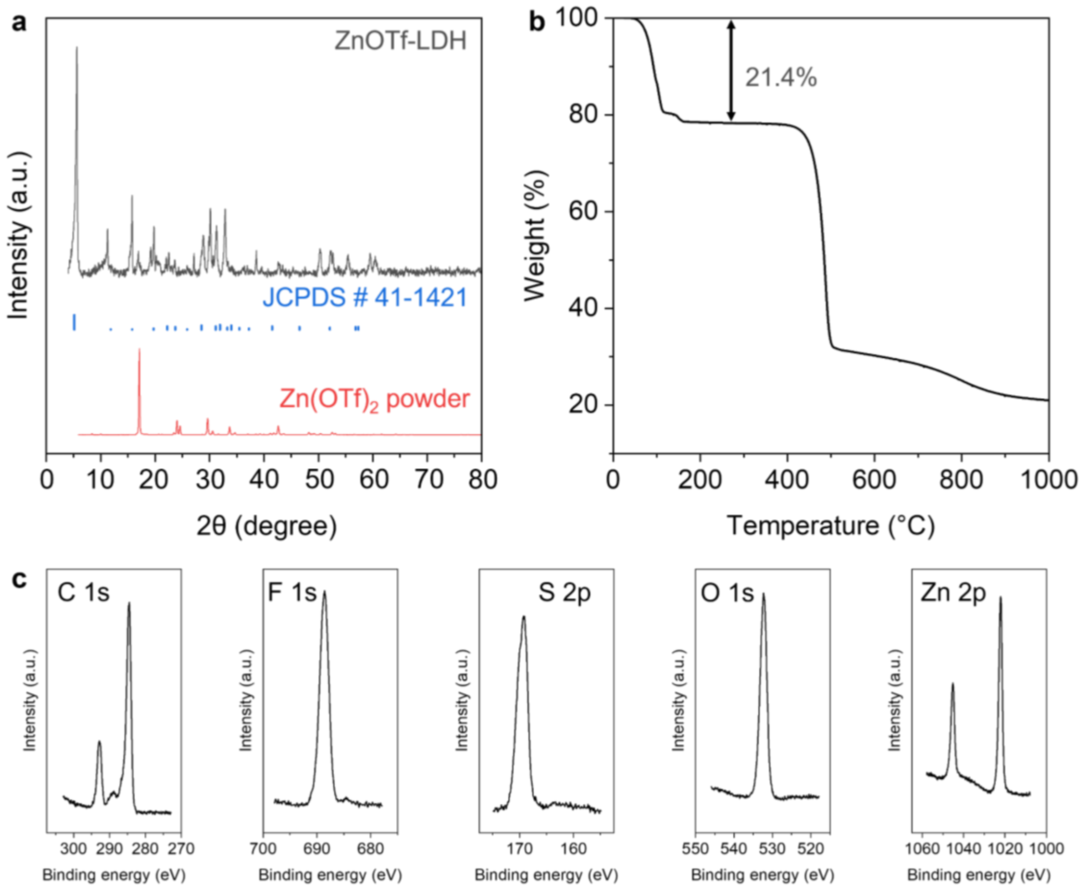

Figure S9. Characterizations including XRD pattern (a), TGA curve (b), and XPS spectra (c) of the as prepared ZnOTf-LDH.

The 21.4% mass loss corresponds to the crystal H_2O in ZnOTf-LDH. This information helps us determine the specific chemical formula of our synthesized ZnOTf-LDH to be $Zn_{12}(OTf)_9(OH)_{15}\cdot 36H_2O$ (referred to Crystallography Open Database, COD ID is 210008), guiding us in establishing the theoretical calculation model.

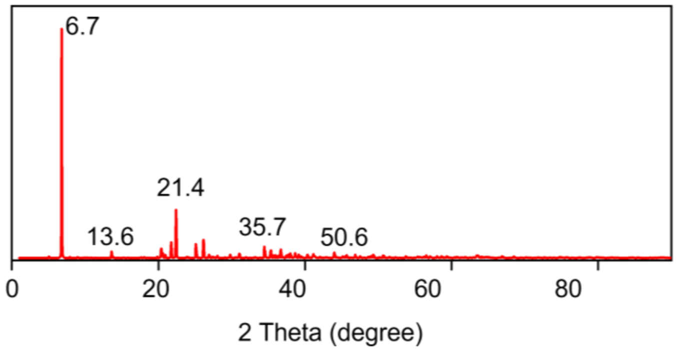

Figure S10. The XRD pattern of our established ZnOTf-LDH calculation model shown in Figure 3 in the main text.

It matches well with our synthesized ZnOTf-LDH as shown in Figure S9 and with the previous literature[3]. Therefore, the model built by our DFT calculation is consistent with the structure in our experiment.

3. Additional calculation details.

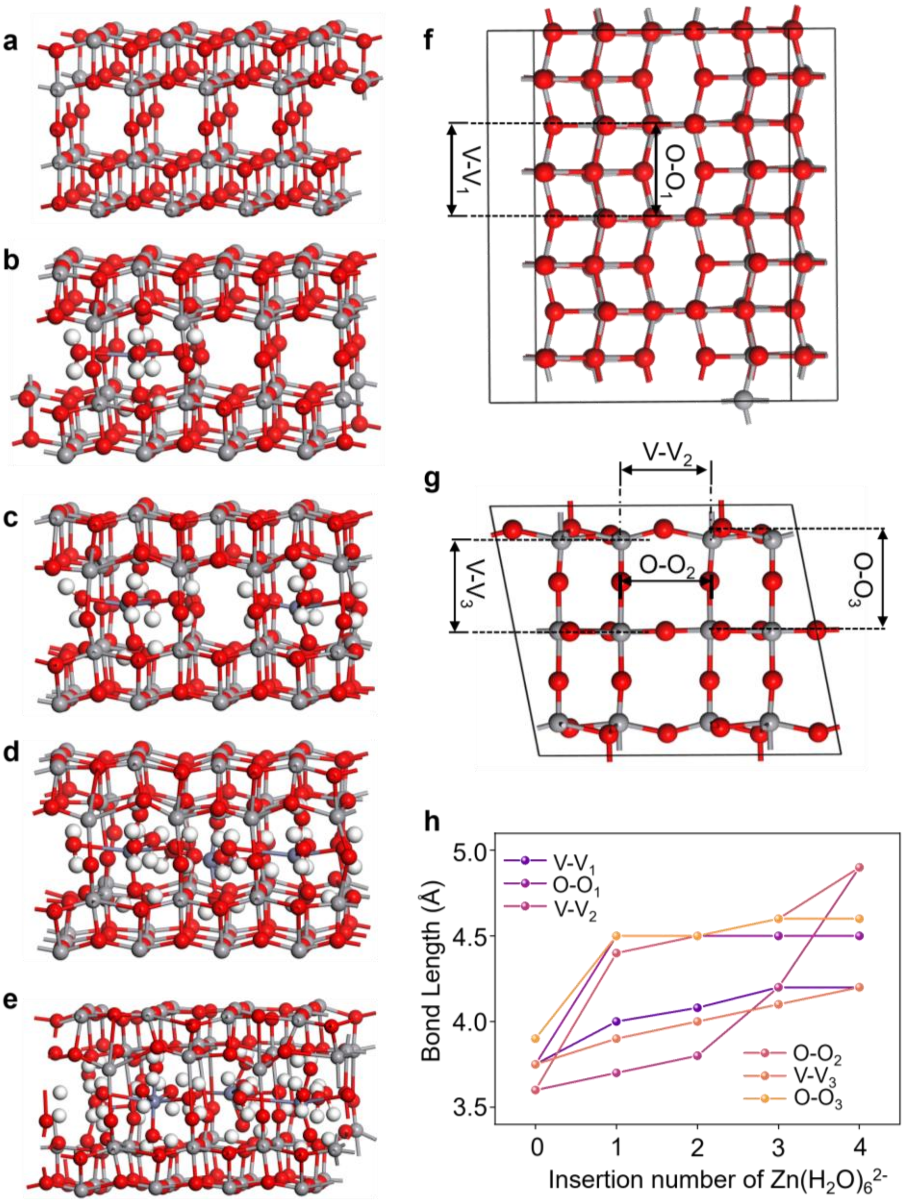

Figure S11. a-e) The structural change upon the insertion of (a) 0, (b) 1, (c) 2, (d) 3, and (e) 4 $Zn(H_2O)_6^{2+}$ in V_6O_{13}. f,g) The V-V_1, O-O_1, V-V_2, O-O_2, V-V_3, and O-O_3 bonds are been labelled for clarity. h) A meticulous bond length analysis.

As depicted in Figure S11, the introduction of $Zn(H_2O)_6^{2+}$ leads to a pronounced elongation of all bond lengths. Notably, a substantial increase in bond lengths is observed for O-O_1, O-O_2, and O-O_3 bonds upon the insertion of a single $Zn(H_2O)_6^{2+}$. The insertion of a second $Zn(H_2O)_6^{2+}$ results in a gradual bond elongation of all bonds, indicating no bond breakage at this stage. However, with the insertion of

the third and the fourth $Zn(H_2O)_6^{2+}$, distinct fractures occur in the V-V_2 bonds. This phenomenon underscores the extensive detrimental effects of $Zn(H_2O)_6^{2+}$ on the cathode materials. Overall, most of the O-O bonds break even with the insertion of only one $Zn(H_2O)_6^{2+}$, and the disruption to the cathode intensifies with the introduction of more $Zn(H_2O)_6^{2+}$, eventually revealing a layered structure. This exposes the dangling bonds of V, making it susceptible to water attack, which leads to significant structural distortions of V_6O_{13} at lower currents.

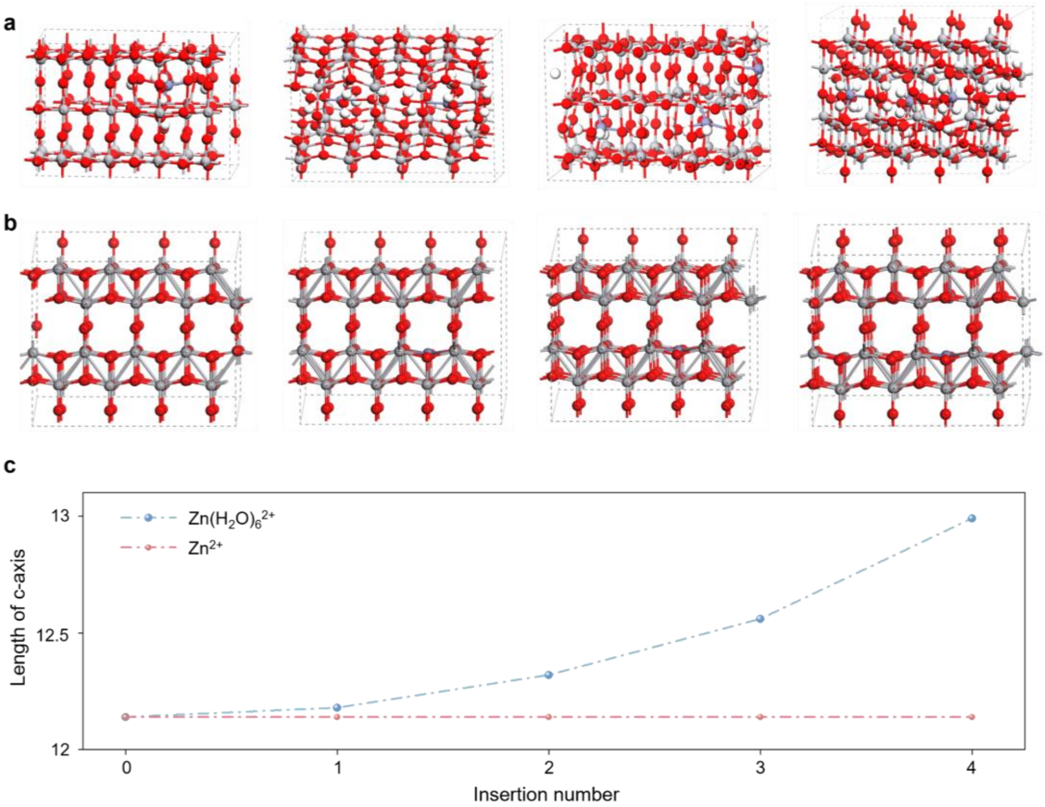

Figure S12. a,b) Changes in the cathode structure with incremental insertion of (a) $Zn(H_2O)_6^{2+}$ and (b) Zn^{2+}. To comprehensively illustrate the structural alterations, we display our models from various perspectives. c) Comparison of the c-axis length variations in the crystal lattice resulting from the insertion of $Zn(H_2O)_6^{2+}$ and Zn^{2+}.

As depicted in Figure S12a, the structure of the cathode material changes significantly during the insertion of $Zn(H_2O)_6^{2+}$, resulting in cathode collapse and impaired long-cycle performance. In contrast, the structure of the cathode material experiences minimal deformation with the increase of Zn^{2+} insertion (Figure S12b), which exhibits good structural stability of the cathode. Figure S12c quantitively substantiates these findings. Upon inserting four $Zn(H_2O)_6^{2+}$ into the cathode material, the c-axis length of the cell expands from 12.1 Å to over 13 Å, corresponding to a minimum volume expansion of 8.3%, which compromises the cathode's stability. As a comparison, the insertion of Zn^{2+} leads to a negligible change in the c-axis length, thus allowing the cathode material to remain stable.

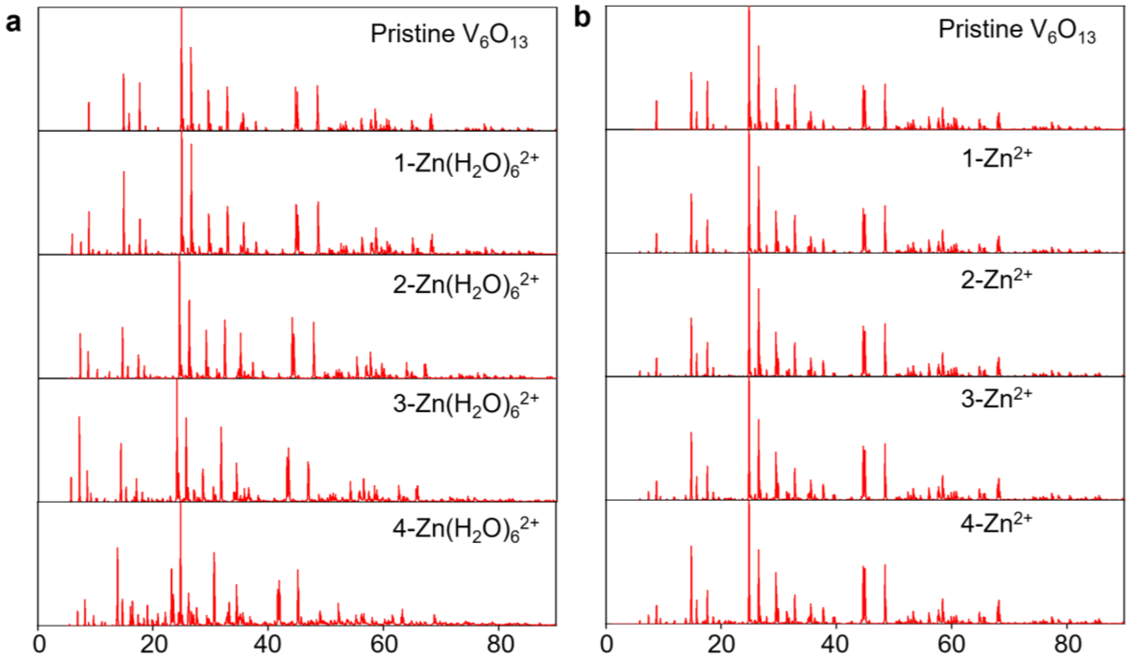

Figure S13. The comparison of the XRD changes when $Zn(H_2O)_6^{2+}$ (a) and Zn^{2+} (a) are inserted into the V_6O_{13}.The insertion of $Zn(H_2O)_6^{2+}$ leads to severe and continuous lattice change of V_6O_{13}, indicating that the suspended bond of V is created at the beginning of intercalation. Besides, with the continuous intercalation of $Zn(H_2O)_6^{2+}$, the deformation of the material becomes increasingly more prominent. Reaction sites of free water and uncoordinated V increase and then intensify dissolution. In the opposite, for the intercalation of Zn^{2+}, the XRD of the material does not undergo any change, indicating that it is difficult for free water to interact with V at this time. Here, the 2×4×1 supercell was constructed to hold the insertion of the Zn^{2+} or $Zn(H_2O)_6^{2+}$, which indicates the $(8\times V_6O_{13})\cdot nZn^{2+}$ or $(8\times V_6O_{13})\cdot nZn(H_2O)_6^{2+}$ (n=1, 2, 3, 4) for the insertion process.

4. Additional comparison of V dissolution behaviors.

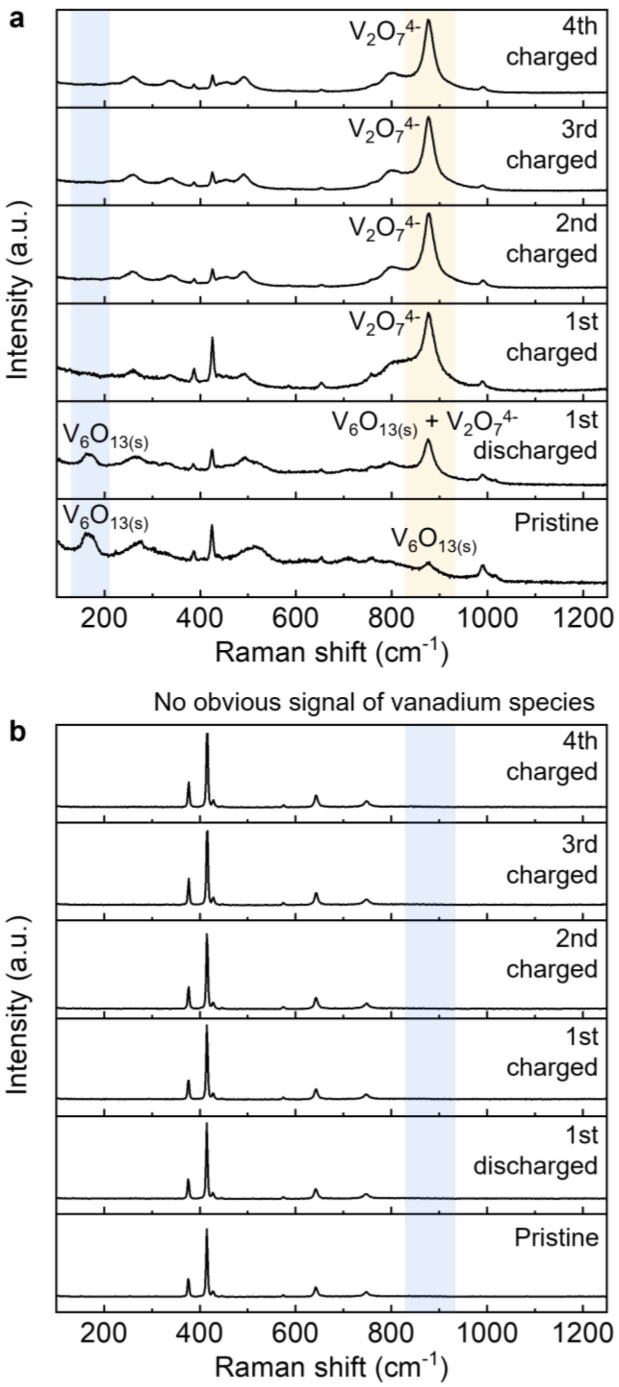

Figure S14. *In situ* Raman test of V_6O_{13} and V_6O_{13}@LDH. Due to the dissolution of vanadium in the V_6O_{13} sample, the $V_2O_7^{4-}$ was detected after cycling, while no signal of $V_2O_7^{4-}$ was detected in V_6O_{13}@LDH. This ion was then combined with the electrolyte to form the $Zn_3(OH)_2(V_2O_7)\cdot H_2O$.

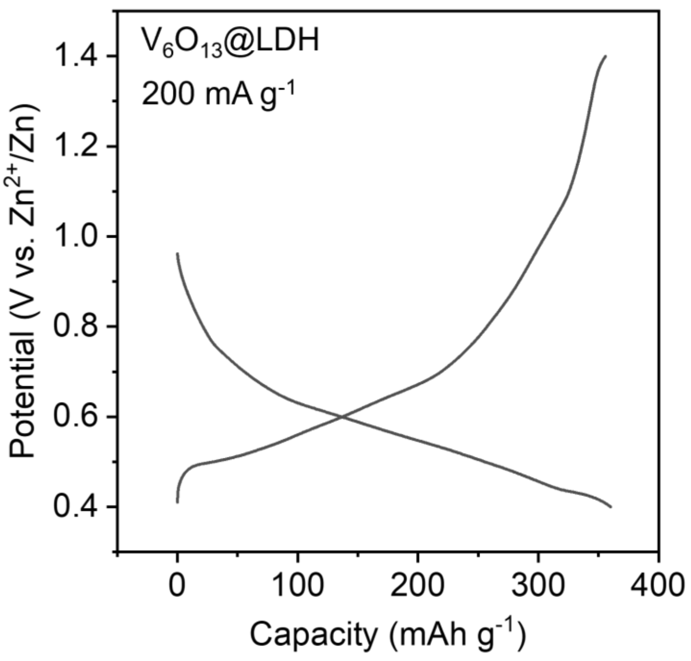

Figure S15. Galvanostatic charge/discharge curve of Zn-V_6O_{13}@LDH cell during the first cycle at a current density of 200 mA g^{-1}. There is no prolonged charging plateau, indicating the inhibited dissolution of vanadium in V_6O_{13}@LDH.

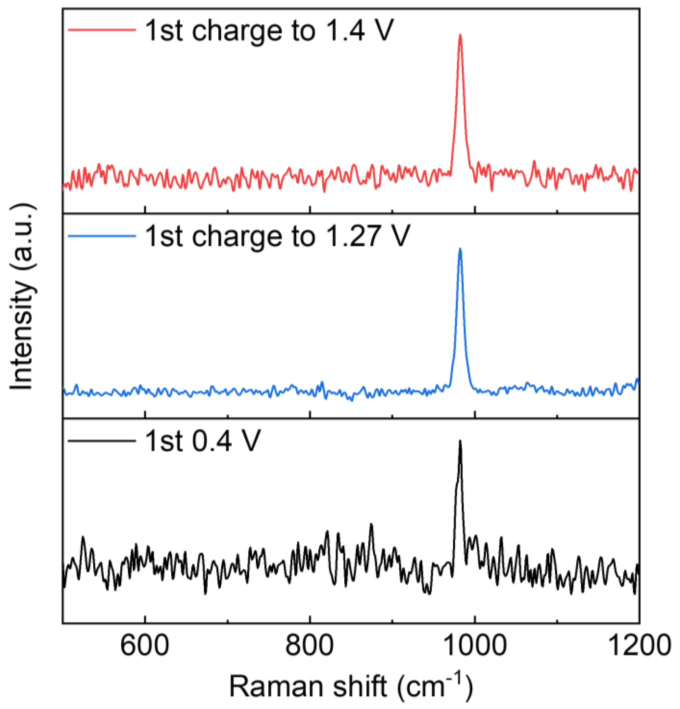

Figure S16. Raman spectra of the Zn foil anode at different charge/discharge states during the first cycle in the Zn-V_6O_{13}@LDH. There is no VO_x signal on the Zn anode surface at the end of the charge, indicating inhibited shuttle of vanadium species.

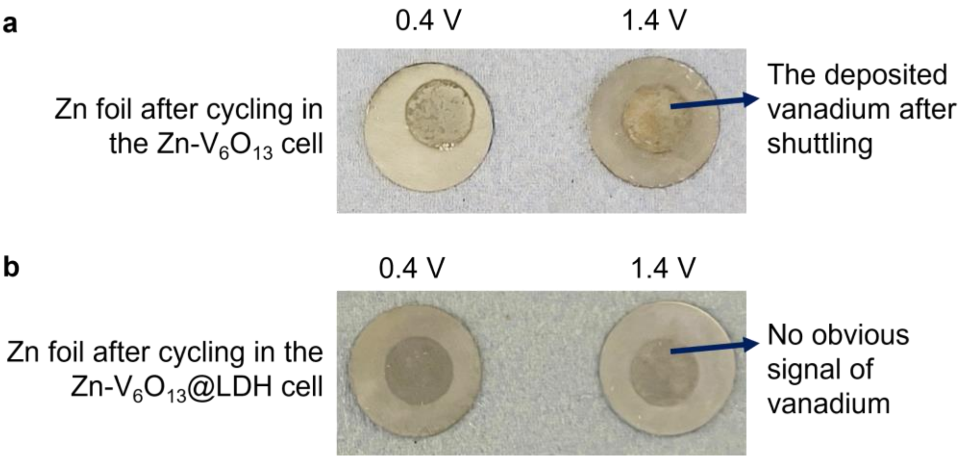

Figure S17. Optical pictures of the Zn anodes after cycling in Zn||V_6O_{13} (a) and Zn||V_6O_{13}@LDH (b) cells at 0.4 V and 1.4 V during the first cycle. The images clearly show vanadium shuttling in the Zn||V_6O_{13} cell, but not in the Zn||V_6O_{13}@LDH cell.

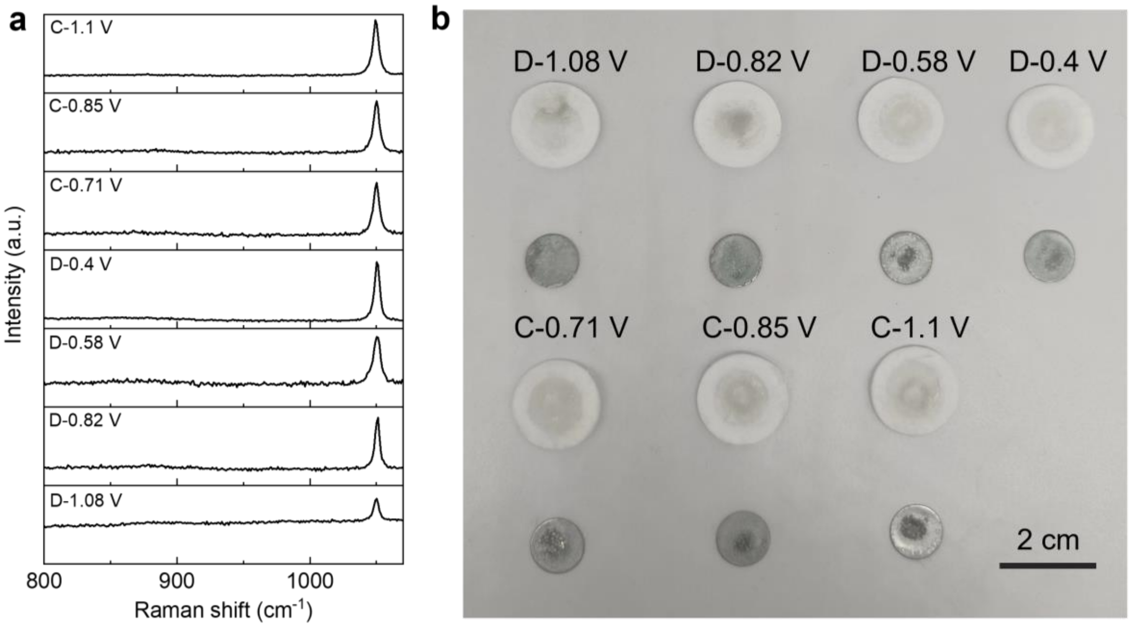

Figure S18. a) Raman spectra and b) optical images of the Zn foil anode and the anode-side separator at different charging/discharging states after cycling in Zn||V_6O_{13}@LDH. The labels beginning with D- were collected during the second discharge process, while those beginning with C- were collected during the first charge process. The cycling was performed at a current density of 200 mA g^{-1}.

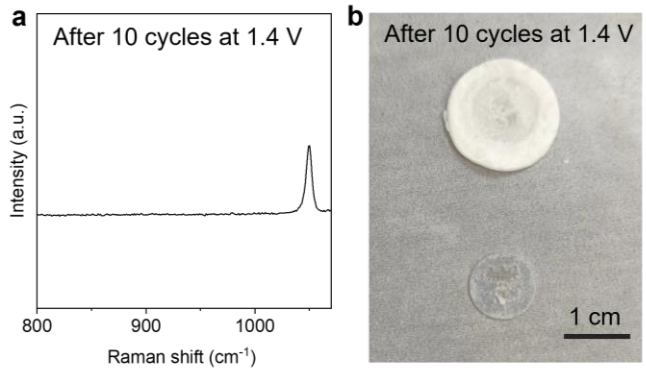

Figure S19. a) Raman spectrum and b) optical images of the Zn foil anode and the anode-side separator at 1.4 V after 10 cycles in Zn||V_6O_{13}@LDH. The cycling was performed at a current density of 200 mA g^{-1}.

As illustrated in Figures S18 and S19, both the initial state and that after 10 cycles demonstrate no apparent formation of earthy yellow precipitates on the zinc foil anode (as observed in Raman spectra and optical images) and the anode-side separator (as observed in optical images) in the Zn||V_6O_{13}@LDH cell. This observation indicates a significant suppression of the dissolution and shuttling of V in the Zn||V_6O_{13}@LDH system owing to the ZnOTf-LDH.

Table S3. Inductively coupled plasma optical emission spectroscopy (ICP-OES) results of the V_6O_{13} and V_6O_{13}@LDH electrodes in their pristine state and after 20 cycles at 200 mA g^{-1}.

	Mass fraction of V element in the electrode-pristine	Mass fraction of V element in the electrode-after 20 cycles	Reduced percentage
V_6O_{13}	15.62%	7.01%	55.1%
V_6O_{13}@LDH	14.57%	11.83%	18.8%

We conducted ICP tests on both the V_6O_{13} and V_6O_{13}@LDH electrodes before and after cycling at 200 mA g^{-1}, specifically focusing on the mass fraction of V for each electrode to enable normalized comparisons. As shown in Table S3, the V content in the V_6O_{13} and V_6O_{13}@LDH electrodes decreased by 55.1% and 18.8%, respectively. The reduction in V content after cycling primarily results from V dissolution within the electrode. These quantitative results demonstrate the effective suppression of V dissolution upon introducing the ZnOTf-LDH interlayer.

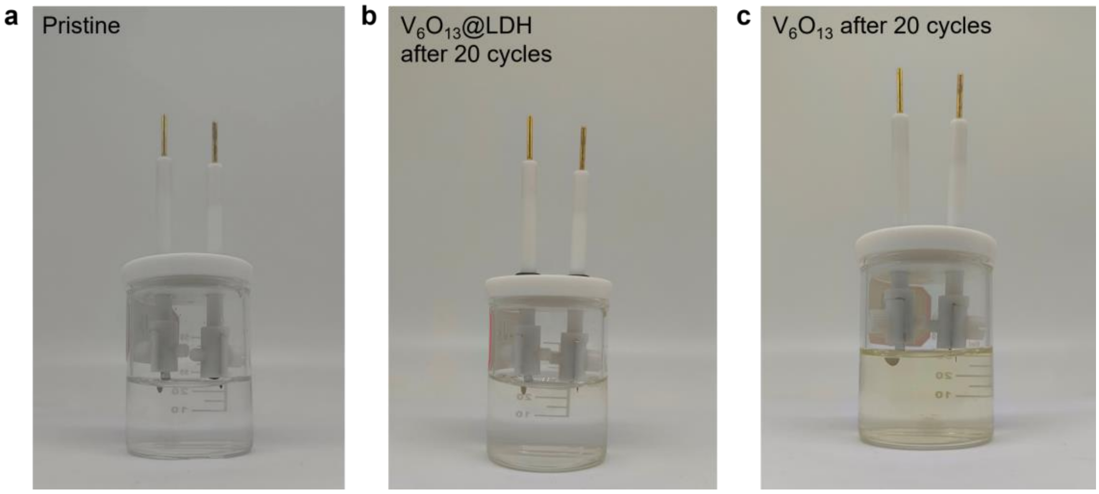

Figure S20. Optical image of the two-electrode cell before and after cycling (at 200 mA g^{-1}) using V_6O_{13} and V_6O_{13}@LDH cathodes. a) Pristine states for both cells. b) Zn||V_6O_{13}@LDH cell after 20 cycles. c) Zn||V_6O_{13} cell after 20 cycles.

As shown in Figure S20, after 20 cycles, the electrolytes of both the Zn||V_6O_{13}@LDH cell and the Zn||V_6O_{13} cell turned yellow, while the degree of yellowing was deeper in Figure S20c. This phenomenon indicates that the V dissolution is more severe in the Zn||V_6O_{13} system, which in turn suggests that ZnOTf-LDH can suppress the V dissolution during cycling.

5. Zn^{2+} storage behavior in the pure ZnOTf-LDH.

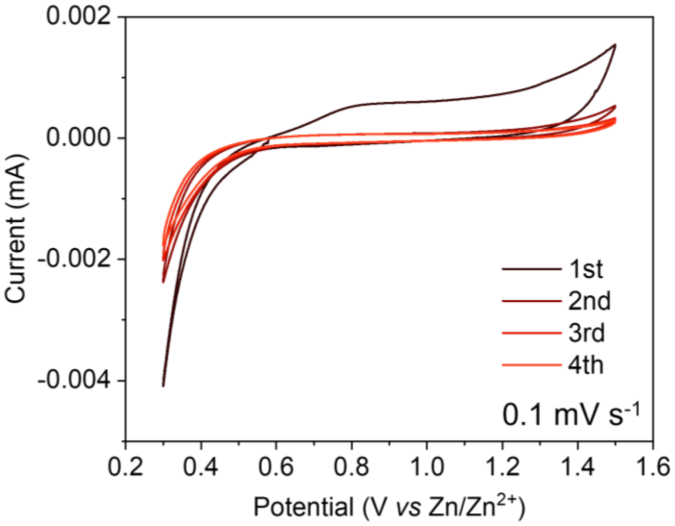

Figure S21. Cyclic voltammogram (CV) curve of Zn||ZnOTf-LDH cell at 0.1 mV s^{-1}. This elucidates that the ZnOTf-LDH does not undergo any redox reactions on its own.

6. *b*-value and analysis of the capacitive current contribution.

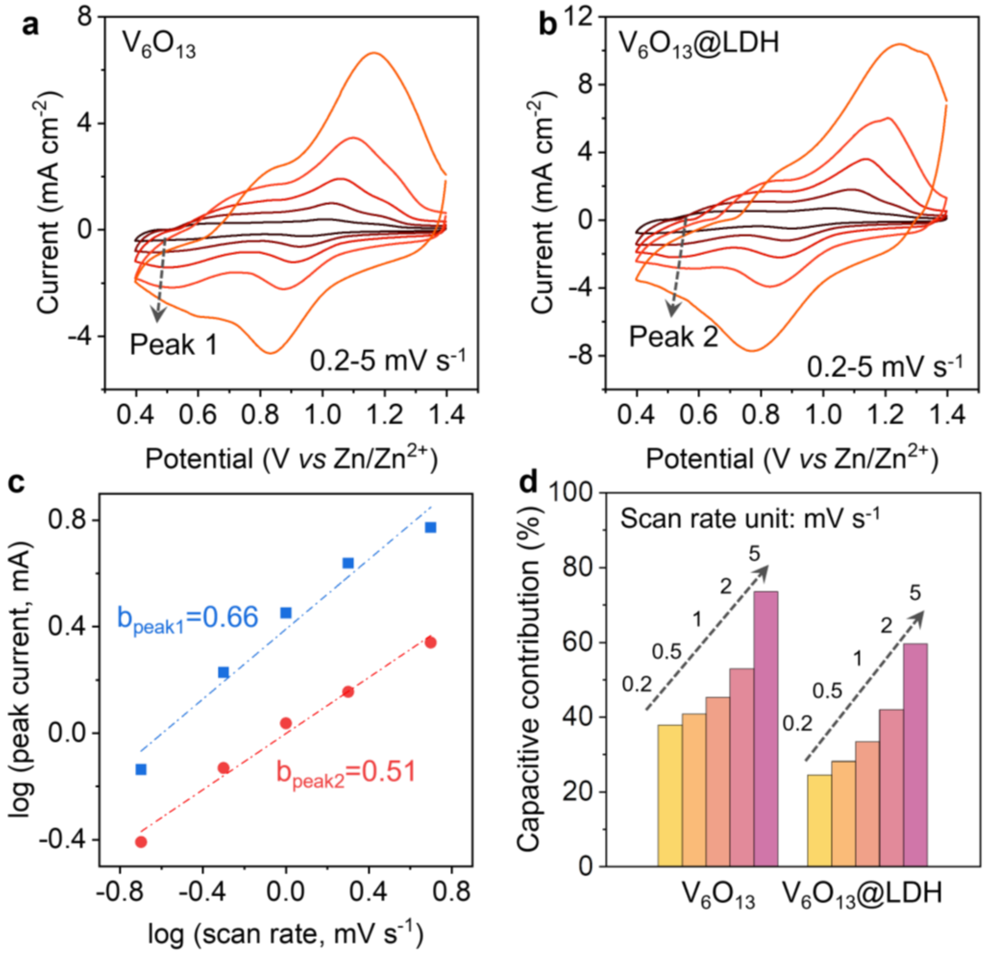

Figure S22. a,b) CV curves of (a) V_6O_{13} and (b) V_6O_{13}@LDH at scan rates from 0.2 to 5 mV s^{-1}. c) Determination of the *b*-value of peak 1 and peak 2 in (a) and (b) by evaluating the relationship between the peak current and the scan rate from 0.2 to 5 mV s^{-1}. d) Ratio of capacitive current to the whole current from 0.2 to 5 mV s^{-1}.

Generally, a *b*-value of 0.5 indicates diffusion-controlled currents, while a *b*-value of 1 corresponds to surface-controlled currents. The *b*-value of the CV peaks associated with Zn^{2+}-species insertion in V_6O_{13} (peak 1) and V_6O_{13}@LDH (peak 2) observed in Figure S22 are 0.66 and 0.51, respectively. These findings, alongside results in the main text, suggest that the charge storage mechanism in V_6O_{13}@LDH likely involves a solid-state Zn^{2+} diffusion process. The deviation of a *b*-value from 0.5 in V_6O_{13} can be attributed to V dissolution reactions originating from the hydrated Zn^{2+} intercalation. This information further enhances our understanding of the electrochemical processes involved in our system.

7. Additional XPS investigations.

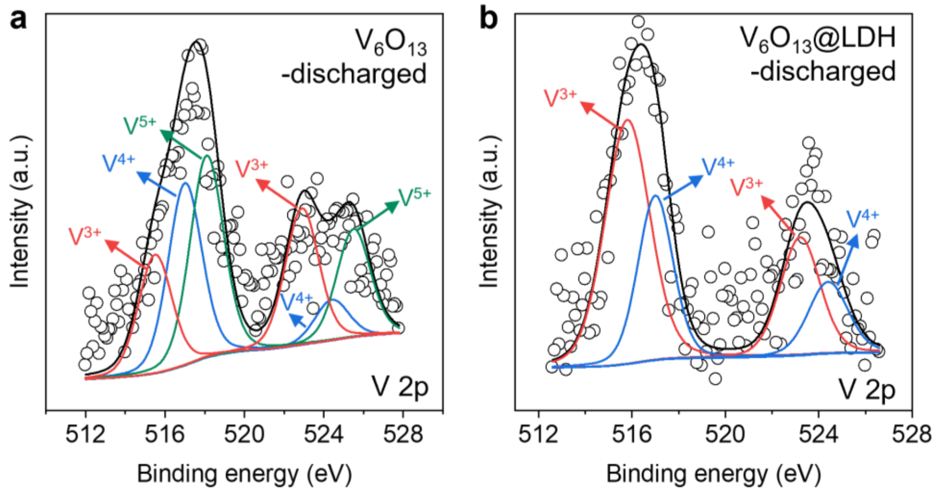

Figure S23. XPS spectra of V 2p core level of (a) V_6O_{13} and (b) V_6O_{13}@LDH in the discharged state. The circle represents the original signal, and the black line represents the fitted curve.

Compared to V_6O_{13} in the discharged state, which consists of V^{3+}, V^{4+} and V^{5+}, V_6O_{13}@LDH in the discharged state consists of only V^{3+} and V^{4+}, indicating a more thorough reduction of vanadium elements in V_6O_{13}@LDH (caused by Zn^{2+} insertion) compared to that in V_6O_{13} (caused by $Zn(H_2O)_6^{2+}$ insertion) after discharge. This observation corresponds to a higher capacity of V_6O_{13}@LDH than V_6O_{13}, as shown in Figure 5a in the main text.

8. Zn||Zn symmetric cell performance.

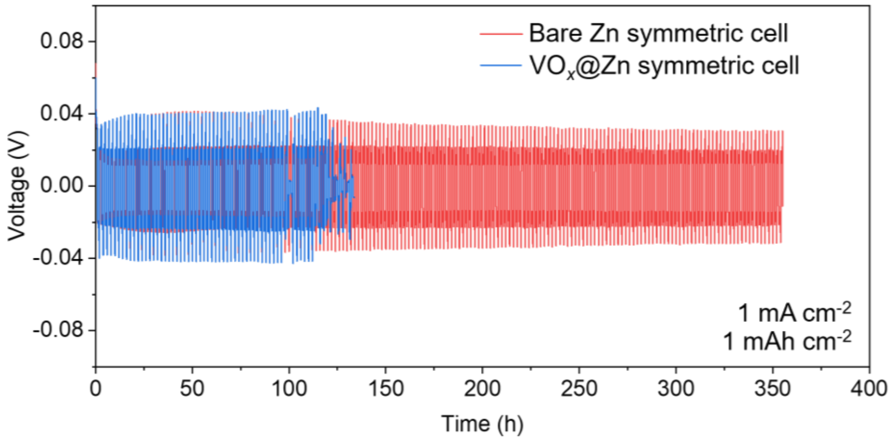

Figure S24. Galvanostatic cycling curves of Zn||Zn symmetric cells at 1 mA cm^{-2} with a fixed capacity of 1 mAh cm^{-2}, using bare Zn and VO_x@Zn electrodes and 3 M $Zn(OTf)_2$ electrolytes.

After cycling in Zn||V_6O_{13} cells, the anodic Zn foils (denoted as VO_x@Zn as V has been shuttled to the anode surface) were collected and further used to fabricate the Zn||Zn symmetric cells. As seen in Figure S24, the cycle life of the VO_x@Zn symmetric cell is shorter than that of the bare Zn symmetric cell, indicating that the shuttled V has contaminated the anode surface, further resulting in a poor cycle life of Zn||V_6O_{13} cells at low current densities.

9. Cycling performance at high current density.

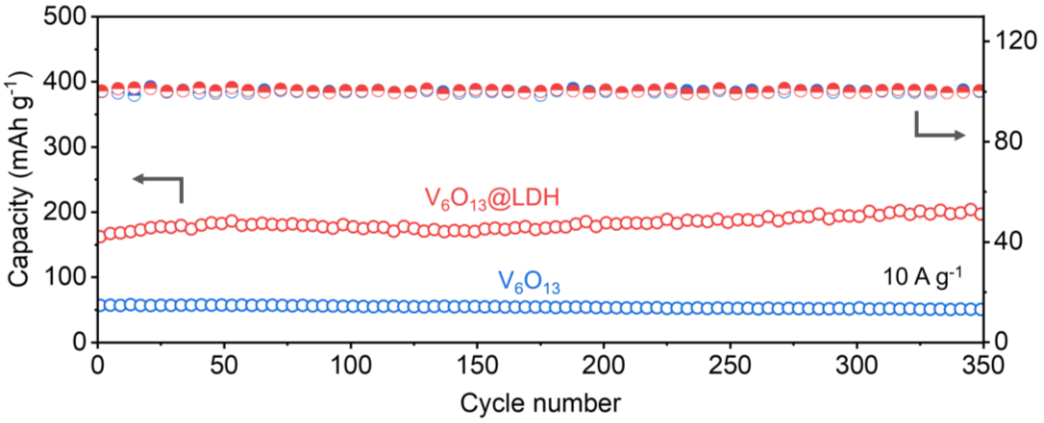

Figure S25. Galvanostatic cycling performance of V_6O_{13} and V_6O_{13}@LDH at 10 A g^{-1}.

At a high current density of 10 A g^{-1}, V_6O_{13}@LDH still delivers stable cycling stability with a higher capacity (180 mAh g^{-1}) than V_6O_{13} (57 mAh g^{-1}).

References

[1] Derek W. Smith, *J. Chem. Educ.* **1997**, 54, 540.

[2] I. Povar, O. Spinu, I. Zinicovscaia, B. Pintilie, S. Ubaldini, *J. Electrochem. Sci. Eng.* **2019**, 9, 75.

[3] L. Wang, K.W. Huang, J. Chen, J. Zheng, *Sci. Adv.* **2019**, 5, eaax4279.
